# Abstract of a Lecture upon Pathology

**Published:** 1873-01

**Authors:** H. R. Noel

**Affiliations:** Professor Physiology and Pathology in the Baltimore College of Dental Surgery.


### ARTICLE III.

### *Abstract of a Lecture upon Pathology.*

BY H. R. NOEL, M. D.,

Professor Physiology and Pathology in the Baltimore College of Dental Surgery.

In our last lecture we decided that this spherical, gelatinous, microscopically minute mass, called *germinal matter* by Beale; *nuclear matter* by Chambers; *proto-plasm* by Huxley, and a *nucleus* by older writers, was in fact the active working agent in the production of all tissues of the body, and assumed the position accorded the *cell* by Virchow. Its powers were three in number, viz: Assimilation, Propagation and Development; by the 1st power, or Assimilation, nutrient fluids were changed into living substance; i. e. vitalized and incorporated with the germinal matter; by the 2nd power, this *germinal matter* could increase in quantity by sub-division of self—by budding and by endogeneous development; by the 3rd power, all tissues were formed, and this was true of vegetable and animal structures alike. With the exhibition of these three powers, we have reached the utmost limit of vitality, and hence we have assigned vitality to the simple manifestation of what are known as the Vegetative Forces. All other phenomena seen in the human organization are, if Physiological, to be ascribed to either Animality, which is simply the working of the animal machinery: muscular, nervous, &c., or to be ascribed to purely chemical and physical causation.

By the "vital forces" the animal machinery is constructed, by the vital forces it is repaired and its integrity preserved, or in other words it is kept in good condition, in good repair; in working order; in a state of *health*, so far as men machinery is involved, and we might say a physiological condition as regards the system, the workings of which evolve Animality.

Unfortunately, however, this spherical mass called "germinal matter," which is the active, working, constructive agent in all histogenetic processes; all tissue building* is liable to perturbations, which most seriously affect its efficiency as an operative. In fact, this restless little microscopic atom is capable of doing as much mischief almost, as it is capable of doing good, and when once started in the wrong direction may give us some most annoying examples of misdirected energy. These deviations from physiological action have been called "Pathological Conditions;" and the products of such deviations are, of course, Pathological Products; but we would expect these products to be at least comparable, if not very nearly the same as the physiological products; *i. e.* the tissues as normally produced. It is therefore wise to begin with the most simple form of deviation from that which is considered normal, and as the normal or physiological condition is the exhibition of all three powers, we will take first that deviation which consists in the abolition or loss of the third or development power.

### FORMATION OF PUS.

When germinal matter has, from any cause, lost the third or developmental, it is prone to take on an inordinate exhibition of the first and second powers. We do not say that this is invariably the case, but that we often observe such a condition to obtain, and the inquiry at once arises as to what products we should expect to find. The answer to this is a production of an excessive amount of *Germinal Matter*—for the vital force seems to expend itself in propagation. Now this excess of germinal matter, with its vitality expended in propagation, is not true and normal tissue; and hence has received a name, characteristic, or at any rate descriptive of it; it is called pus.

Pus is therefore, anatomically considered a mass, or aggregation of germinal matter; which shows no disposition to exhibit any further evidence of vitality than mere assimilation and propagation. If we place such a mass under the microscope, it appears to be composed of globules, of about the same size, form, contour etc.; with here and there yellowish dots, called oil globules; and these oil globules are characteristic of absolute loss of all vital force, and result from a species of chemical decomposition, showing incipient death of globules, and the rapid invasion of chemical forces upon the ebbing vital force. Now as the fatty degeneration advances, more and more of oily matter is found; hence the older the pus, the less the vital force, and the more evident becomes this degeneration. We may therefore expect to find much oily matter, and but little vitality in large masses, of what we shall call pus.

For the present, it is only necessary for you to remember that pus is a product which results from complete abolition of the developmental power of germinal matter, with a decided tendency to exhibition of the assimilative and propagative. This very strange abolition of one power, or exhibition of vital force, could only occur in consequence of devitalization of germinal matter; and will of course be found more frequently in local or general adynamics of special tissues, or the system as a whole. Boils, abscesses, scrofulous ulcers, tubucular caverns, etc., are examples of lower adynamical conditions, with the formation of this pathological product.

### MUCUS AND FIBRIN.

In some instances, this lowering of the vital powers does not reach the point of complete abolition of the third or developmental expression of force, and we may have only a partial arrest of this third power. In such a condi tion the power, though manifested, would be in an abortive form; and though we should not have pus, we would have another product, which was not true tissue; but an irregular, abnormal, or pathological product.

This element would be one of partial arrest of development, and must therefore differ from pus where the arrest has been complete. We might suppose that the causation was of the same kind,but differing in intensity, and that when very intense, *pus* would result; but when not so intense, the abortions of the third or developmental power would be found.

This is true, and we have in the carmine test of Lionel S. Beale, a means of diagnosing between the simple pus formations, and the formations of higher, though abortive products of germinal matter; in the former the whole mass is stained red, as you remember germinal matter to have been; in the latter class, only the very few and accidentally retained masses of germinal matter are stained, while the imperfect manifestations of the third or developmental power remain unchanged. Of this abortion of the third power, or partial arrest of it, *mucus*, *fibrin*, and the *amyloid change* are our best examples.

The description given of mucous formation by Chambers, Virchow, and others, when sharply compared, will show the accuracy of the above statements.

If now we compare the statements made by Paget, Buchanan, A. Schmidt, Virchow, L. S. Beale, and Todd & Bowman, we can readily harmonize them upon this fact, that fibrin is of local formation, found in tissues and in blood alike; and formed from the white blood corpuscle, and from connective tissue corpuscles, and is most rapidly formed when there is lowered vitality; *i. e.* partial arrest of third or developmental power.

We shall therefore place fibrin with mucus, as a pathological element, and also as one most liable to be formed in depraved and depressed conditions of the system. Thus in inflammations, such as pleurisy, pneumonia, erysipelas, etc., we should expect to find fibrin, and as a fact, we do find it in large quantities. In many cases of purulent expectoration accompanying tubercular consumption, we can demonstrate under the microscope the presence of pus, mucus, and fibrin, in the same mass; thus showing their closely allied origin, and this goes far towards establishing their relation to germinal matter.

If a small area of connective tissue corpuscles, immediately beneath the skin, be acted upon by this depressing agent, we may have one, or if more areas be involved, have several local formations of pus; and we call these circumscribed formations pus *boils*.

If the germinal matter of the skin be acted upon by such an agent, as for example the very intense heat of boiling water, etc., we have a superficial pus formation in the suppurating surface burned. If an extensive area of connective tissue corpuscles be involved, as for instance in the forearm, the product may not be pus, but a formation of fibrin, as is seen in what we call diffused phlegmon, or phlegmonous erysipelas. Here the formation is so very rapid that the skin is pushed out from the structures beneath, by this fibrin, and the arm appears swollen, with a mottled arrangement of white and red, and sometimes almost a complete white, waxy color. The waxy whiteness is characteristic of great distension and shows a pressure so great as to obliterate temporarily the capillaries of the skin. The layer of fibrin, may be from one-half to an inch thick, or more.

Now, when the same rapid, local formation of fibrin occurs in the lungs, we call it *pneumonia;* if in the liver, we call it hepatitis; if in the kidneys, a nephritis, etc.; the name varying with the organ attacked; but the true condition is the *one* thing; *i. e.* the formation of fibrin from deviations in the exhibition of the third or developmental power of germinal matter.

In a few instances, mucus is formed on the skin, in diseased bone, and muscular tissue; but its most frequent position is upon the free epithelial surface of mucous membranes.

Upon this free epithelial surface we also find pus, as in purulent catarrh, laryngeal and pharyngeal troubles, and in some of the graver ophthalmias. Here also we find fibrin in membranous croups, diptherias, etc. From these facts, you will at once perceive the close relationship of pus, mucous, and fibrin, as pathological products; and now appreciate the fact that the misdirected energy of the corpuscle, or mass of germinal matter, is indeed potent for evil.

*To be continued.*

